# A Novel and Reproducible Urinary Diagnostic Framework Reduces Health Care and Antibiotic Utilization for Urinary Tract Infections

**DOI:** 10.1093/ofid/ofaf293

**Published:** 2025-05-15

**Authors:** Kendall Kling, Teresa Zembower, W Justin Moore, Janna Williams, Amanda Vo, Stephanie Colbert, Anthony Schaeffer

**Affiliations:** Division of Infectious Diseases, Department of Medicine, Feinberg School of Medicine, Northwestern University, Chicago, Illinois, USA; Department of Pathology, Feinberg School of Medicine, Northwestern University, Chicago, Illinois, USA; Division of Infectious Diseases, Department of Medicine, Feinberg School of Medicine, Northwestern University, Chicago, Illinois, USA; Department of Pathology, Feinberg School of Medicine, Northwestern University, Chicago, Illinois, USA; Department of Antimicrobial Stewardship, Northwestern Medicine, Chicago, Illinois, USA; Division of Infectious Diseases, Department of Medicine, Feinberg School of Medicine, Northwestern University, Chicago, Illinois, USA; Department of Urology, Feinberg School of Medicine, Northwestern University, Chicago, Illinois, USA; Department of Urology, Feinberg School of Medicine, Northwestern University, Chicago, Illinois, USA; Department of Urology, Feinberg School of Medicine, Northwestern University, Chicago, Illinois, USA

**Keywords:** antibiotic stewardship, asymptomatic bacteriuria, multidrug-resistant bacteria, urinary tract infections, UTI diagnostic framework

## Abstract

**Background:**

An interdisciplinary clinic including specialists in infectious diseases, urology, and antimicrobial stewardship developed a standardized urinary infection diagnostic framework (UDF) for patients referred for complex urinary tract infections (UTIs).

**Methods:**

We performed a descriptive review and evaluation of an interdisciplinary UTI clinic utilizing a standardized UDF, tracking outcomes such as infection management and resolution, health care utilization, and antibiotic exposure prior to and following the initial clinic visit. The UDF leveraged anatomic categorization (complicated or uncomplicated) and diagnostic categorization via specific symptom and culture criteria. Patients were treated according to their diagnostic categorization, including supportive care, UTI treatment and prevention therapies, and surgery, if appropriate.

**Results:**

A total of 216 patients were referred for complex UTIs. Sixty-eight (32%) patients referred for UTIs were found to have noninfectious syndromes. Among 70 patients with recurrent infections undergoing prevention therapy, 69% were UTI free at a mean follow-up of 4.6 months (range, 2–13). In the 20 patients with urinary bacterial persistence, a urologic nidus was identified, and a multimodal approach managed the infection in 95% (eg, removal of source or antimicrobial suppression). As compared with 1 year before the initial clinic visit, patients required fewer hospitalizations (*P* < .001), emergency department/urgent care visits (*P* < .001), and antibiotic courses and days of therapy (*P* < .001) for UTIs 1 year after the initial clinic visit or 1 year after source control for persistence.

**Conclusions:**

An interdisciplinary UDF standardizes management of urinary complaints and can reduce hospitalizations, emergency department/urgent care visits, and antibiotic use for UTIs.

## Teaser

A urinary infection diagnostic framework within an interdisciplinary clinical setting clarifies diagnoses, standardizes management, and reduces health care utilization and antibiotic use for patients referred for urinary tract infections.

Urinary tract infections (UTIs) are the most common bacterial infections in humans, annually affecting >150 million people worldwide and leading to an estimated cost of $3 billion to $5 billion dollars in the United States [1–5]. Urinary complaints are among the most frequent concerns in acute care and ambulatory settings [2–6]. In clinical practice, patients referred for urinary symptoms constitute a wide spectrum of conditions, including noninfectious syndromes, isolated or intermittent infections, recurrent infections, or urinary bacterial persistence, which can present challenges to clinicians. In addition, the definition of complicated UTI has undergone many revisions, leading to clinician ambiguity and confusion on how to optimally diagnose and treat patients with complex urologic anatomy [6–8].

Asymptomatic bacteriuria (ASB) and noninfectious lower urinary tract symptoms (LUTS) pose significant conundrums for clinicians across a broad spectrum of specialties, often due to the multitude of symptoms that are mistakenly attributed to UTIs, including foul-smelling urine, cloudy urine, fatigue, malaise, back pain, and incontinence, among others [9, 10]. The Infectious Diseases Society of America (IDSA) currently recommends treating ASB only in the setting of pregnancy, for an upcoming urologic procedure involving mucosal disruption, or within the first month after kidney transplant [11]. Despite this, many patients with ASB receive antibiotics, contributing to the growing rates of multidrug-resistant organisms (MDROs) [12–19].

Recurrent infections, defined as 2 infections in a 6-month period or ≥3 infections in a year, are observed in 30% to 50% of women with UTIs and have been found to have a negative impact on mental health, yet effective prophylactic measures may be underutilized in up to 40% of patients [20–22]. Some patients with recurring infections may have bacterial persistence that requires urologic evaluation to identify and remove a focus of infection within the urologic tract, but there are no formal guidelines recommending imaging studies for patients with UTIs [23, 24].

Our institution established a Complex Urologic Infections Interdisciplinary Clinic, where specialists in infectious diseases, urology, and antimicrobial stewardship jointly evaluate patients utilizing a novel urinary infections diagnostic framework (UDF) to standardize the diagnosis and management of UTIs.

## METHODS

This study was approved by the Northwestern Institutional Review Board (STU00221676).

**Figure 1.**
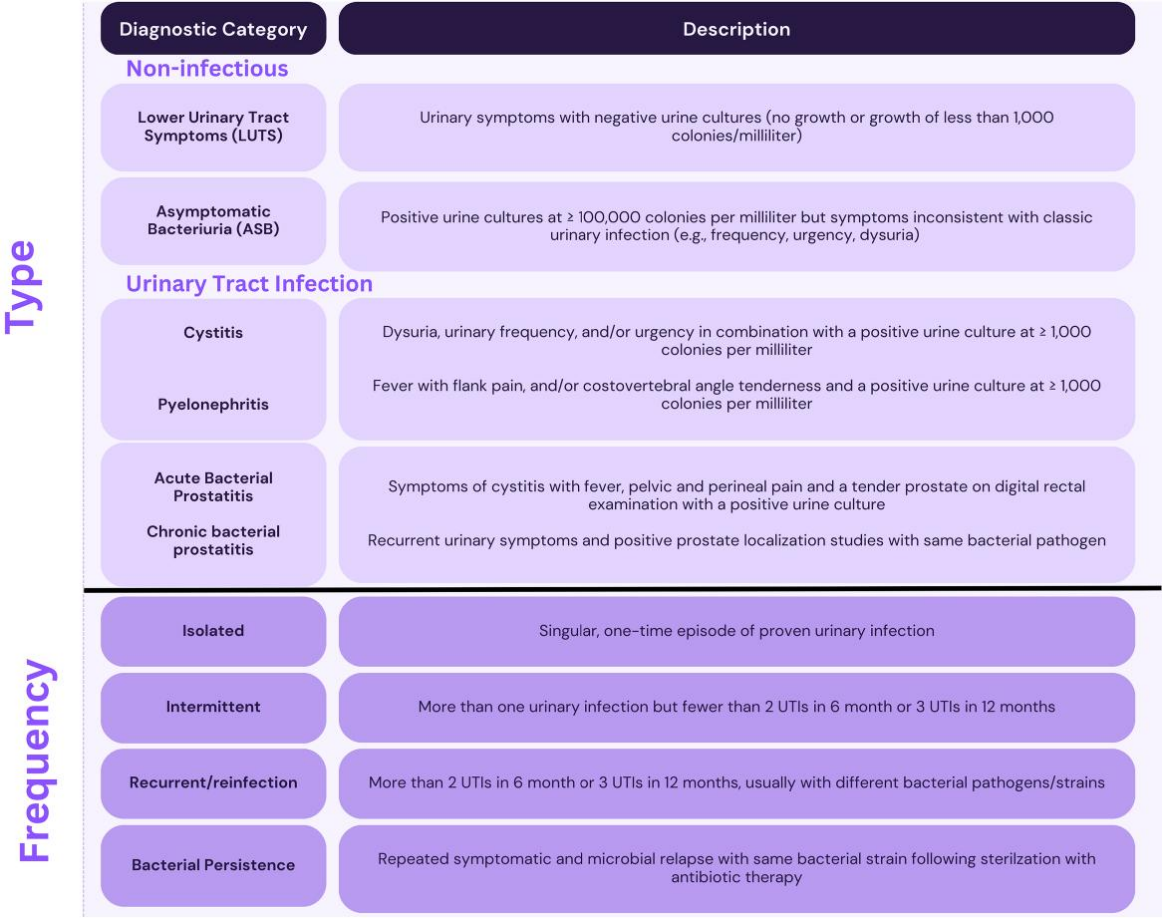
Urinary infections diagnostic framework. UTI, urinary tract infection.

Patients were referred to the Complex Urologic Infections Interdisciplinary Clinic for concern for complex UTIs, and referrals came from urologists, primary care providers, urgent care and emergency medicine providers, infectious diseases specialists, other subspecialty referrals, or self-referrals. All patients were seen by infectious diseases specialists (2 of whom have joint appointments in clinical microbiology), and all cases were discussed and categorized via the UDF and group consensus in a same-day interdisciplinary meeting with infectious diseases specialists, urologists, and antimicrobial stewardship pharmacists (Figure 1). Antimicrobial stewardship pharmacists trained in infectious diseases assisted in selecting agents for MDROs, perioperative antibiotic dose and timing, and drug-drug interaction considerations. The clinic opened 6 October 2022, and the first 237 patients seen in the clinic were included in this review. Outcomes data were collected as a retrospective cohort study by study team investigators.

### Evaluation

Each visit included documentation of the patient's history, including symptoms during a UTI event, antibiotic treatment record, symptom response to treatment, prior prevention strategies, and prior urologic abnormalities and surgery. Available urine culture data were reviewed for organism patterns and quantification, and subsequent events thought to represent UTI were documented by urine culture. All patients referred to the clinic had postvoid residual urine measured as part of routine workup.

### Categorization

Cases were first classified as either complicated or uncomplicated based on anatomic urinary tract structure and function, assessed per history and diagnostic studies when appropriate. Complicated cases featured an abnormal urinary tract structure or function that was judged to contribute to the development of infections.

Cases were further categorized into descriptive diagnostic categories that were standardized as outlined in Figure 1. Of note, we did not consider suprapubic pain, altered mental status, fatigue, incontinence, abdominal pain, nausea, urinary retention, cloudy urine, or foul-smelling urine to be reliable symptoms of UTIs based on the current literature citing poor sensitivity and specificity [14, 17–19, 25, 26]. Also, we did not consider painless microscopic or macroscopic hematuria without other symptoms of infection (as listed in the framework) to be diagnostic of UTI, although we did refer for urologic evaluation if found [27]. Some patients could not reliably communicate LUTS, such as patients who were nonverbal; patients with indwelling urinary catheters, suprapubic catheters, ileal conduits, or neobladders; and patients performing intermittent urinary catheterization. In that population, patients were considered to have UTIs only if they developed fever with leukocytosis or septic shock of no other known cause with a concurrent positive urine culture result at ≥1000 colonies/mL. For patients with spinal cord injury, we did not consider symptoms such as bladder spasm, muscular spasticity, worsening dysreflexia, and change in voiding habits as reliable markers for UTI given the low specificity in this population [28].

Last, although we did order urinalyses for our patients, we did not consider the presence or absence of leukocytes, nitrites, or leukocyte esterase a part of the definitive diagnostic criteria for UTIs. If a patient had symptoms consistent with cystitis and concurrent supportive culture results, we designated that as a UTI event, regardless of the urinalysis. Conversely, if a patient had symptoms inconsistent with UTI (eg, foul-smelling urine) but had leukocytes, we would not consider this a UTI event, due to literature showing that most patients with ASB have pyuria [29]. In this regard, we used the urinalysis as initial guidance for those with symptoms but not as the gold standard diagnostic test. If abnormalities were discovered with negative cultures (eg, proteinuria, microscopic hematuria), we would consider appropriate referral.

### Clinical Management Based on the UDF

Cases of isolated or intermittent infections were managed with standing orders for urinalysis and urine culture with treatment as needed. Patients with recurrent/reinfection were offered prevention therapy, such as vaginal estrogen, postcoital prophylaxis, and/or continuous prophylaxis. Self-start antimicrobial therapy based on prior urine culture susceptibilities was provided to patients with recurrent infections who declined preventative measures; specifically, a standing prescription order was to be filled upon the development of symptoms within the UDF. These patients were instructed to first provide a urine sample for urinalysis and culture prior to initiating self-start therapy. Patients with bacterial persistence underwent imaging of the urologic tract (eg, ultrasound, computed tomography, and/or appropriate bacterial localization studies) to attempt to identify an infected source. If a urinary tract abnormality was discovered, we referred the case to the urology department for management. Surgical prophylaxis and other referrals were treated individually.

### Outcomes

Demographic information, clinical outcomes, efficacy of prophylaxis, MDRO data, adverse effects of antibiotics, surgical interventions, and antibiotic utilization were determined by follow-up visit and electronic medical record review. The primary outcome was to evaluate the impact of the UDF as measured via hospitalizations, emergency department/urgent care visits, and antibiotic utilization for UTIs, including incomplete courses, 1 year prior to and 1 year after initial clinic evaluation or, for patients with bacterial persistence, 1 year from the date of source control. The secondary outcome was prophylaxis efficacy via the presence of UTI at any point after the initial clinic visit, as determined via follow-up visit to the clinic. Note that the study was not powered to compare differences in prophylaxis regimens for recurrent UTIs. Paired *t* test analysis to compare means and χ^2^ analysis to compare proportions were performed in Microsoft Excel.

## RESULTS

### Participant Characteristics

Demographics (sex, race, and comorbidities) were obtained via the electronic health record. Most patients self-identified as female sex (69%) and White race (67%), with a mean age of 61 years (range, 19–94) and a low mean Charlson comorbidity score of 3.3 (Supplementary Table 1). The most common comorbidity was cancer (29%; mostly prostate and bladder), followed by diabetes (19%) and chronic kidney disease (9%). The mean follow-up from the initial clinic visit was 9.8 months, with a range of 3 to 16 months. Forty percent of patients had a follow-up appointment within the study period, with most having 1 follow-up appointment (70%), followed by 2 (18%), 3 (8%), and 4 (3%) appointments.

### Anatomic and Diagnostic Categorization of Participants

Most patients (58%) were deemed to have complicated urologic tracts (Supplementary Table 2). Of the 73 male patients, 57 (78%) were designated as having a complicated urinary tract, in contrast to 80 (49%) of the females. Patients with complicated urologic tracts had significantly higher Charlson comorbidity scores, rates of prostate and bladder cancer, and rates of hemiplegia and were more likely to present with recurrent/reinfection and pyelonephritis.

**Figure 2.**
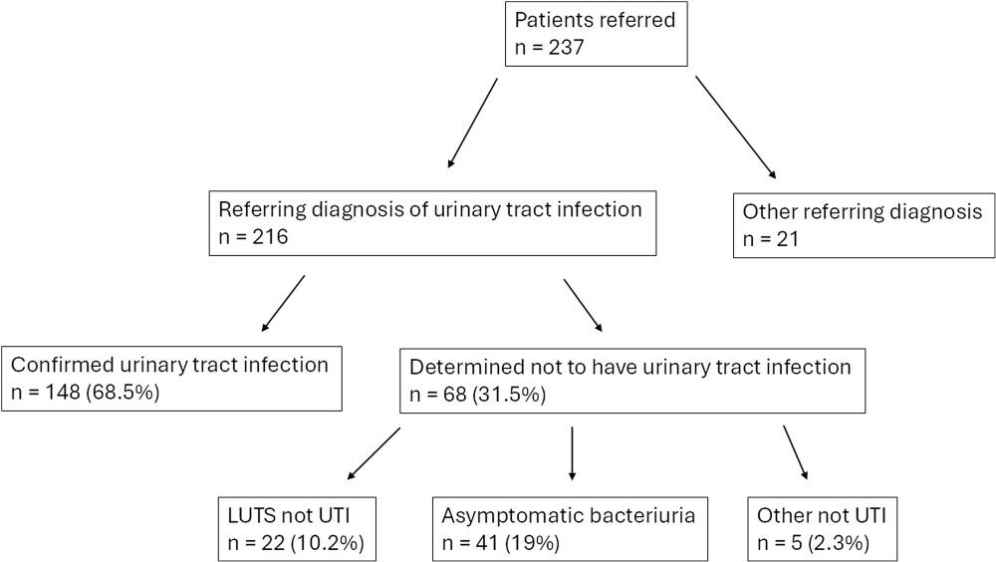
Categorization of patients referred by the urinary diagnostic framework. “Other not UTI” included 3 patients with hematuria, 1 with sterile pyuria, and 1 with flank pain. LUTS, lower urinary tract symptoms; UTI, urinary tract infection.

Clinician review leveraging the UDF sorted cases into diagnostic categories (Table 1). Of the 216 patients initially referred with a diagnosis of UTI, 68 (32%) were determined not to have a symptom and/or true culture-proven UTI at any point (Figure 2). The most common complaints mistaken for UTI symptoms were incontinence, foul-smelling urine, urine color/consistency change, fatigue, vaginal irritation/itching/burning, testicular pain, abdominal pain, back pain, and incomplete voiding sensation. Of the 148 patients with confirmed UTIs, 9% had isolated UTI (mostly pyelonephritis, 59%), 7% had intermittent UTI (mostly cystitis, 77%), 38% had recurrent/reinfection (mostly cystitis, 64%), and 8% had bacterial persistence.

**Table 1.** Categorization of Participants Using the Urinary Infections Diagnostic Framework

	All Patients (n = 237)	Complicated (n = 137)	Uncomplicated (n = 100)	*P* Value
Noninfectious syndromes (n = 68)				
Asymptomatic bacteriuria	41 (17.3)	20/41 (48.8)	21/41 (51.2)	.81
LUTS not UTI	22 (9.3)	4/22 (18.2)	18/22 (81.8)	<.001
Other non-UTI^a^	5 (2.1)	0/5 (0)	5/5 (100)	<.001
Isolated UTI	22 (9.3)	13/22 (59.1)	9/22 (40.9)	.069
Cystitis	7 (31.8)	2 (15.4)	5 (55.6)	<.001
Pyelonephritis	13 (59)	10 (76.9)	3 (33.3)	<.001
Prostatitis	1 (4.5)	0	1 (11.1)	<.001
Epididymo-orchitis	1 (4.5)	1 (7.7)	0	.008
Intermittent UTI	17 (7.2)	8/17 (47.1)	9/17 (52.9)	.56
Cystitis	13 (76.5)	5 (62.5)	8 (88.9)	.03
Pyelonephritis	4 (23.5)	3 (37.5)	1 (11.1)	<.001
Recurrent/reinfection	89 (38.0)	54/89 (60.7)	35/89 (39.3)	.032
Cystitis	57 (64)	26 (48.1)	31 (88.6)	.001
Pyelonephritis	31 (34.8)	27 (50)	4 (11.4)	<.001
Acute prostatitis	1 (1.1)	1 (1.9)	0	.168
Bacterial persistence	20 (8.4)	19 (95)	Unknown	…
Source identified	19 (95)	…	…	
Stone	10	10	0	
Bladder sling	1	1	0	
Chronic prostatitis	2	2	0	
Urethral sling	1	1	0	
Emphysematous pyelonephritis	1	1	0	
Retained surgical wire	1	1	0	
Artificial urinary sphincter	1	1	0	
Urethroplasty	1	1	0	
Cystitis cystica	1	1	0	
Source unidentified	1 (5)	Unknown	Unknown	…
Surgical prophylaxis	15 (6.3)	14/15 (93.3)	1 (6.7)	<.001
Other^b^	6 (2.5)	4/6 (66.7)	2/6 (33.3)	.001

Data are provided as No. (%).

Abbreviations: LUTS, lower urinary tract symptoms; UTI, urinary tract infection.

^a^Including 3 patients with hematuria, 1 with sterile pyuria, and 1 with flank pain.

^b^Including 3 patients with imaging abnormalities (nephrolithiasis, air in bladder after cystoscopy, and hydronephrosis), 2 with pubic symphysis osteomyelitis, and 1 with chlamydia.

### Isolated or Intermittent UTI Outcomes

Of the 22 patients with isolated UTI, only 1 (5%) had a UTI at 2 months (mean follow-up, 9 months; range, 6–14). Of the 17 patients with intermittent UTI, 3 (18%) had a breakthrough UTI at a mean 3.6 months (mean follow-up, 8 months; range, 5–14).

### Recurrent/Reinfection Outcomes

Among 89 patients with recurrent/reinfection, 84 were included in the analysis of prevention efficacy (the remaining 5 were undergoing inconsistent multimodal forms of prevention). Of these 84, 55 (66%) were UTI free at follow-up at a mean 4.7 months (range, 2–13). Of the 14 patients who declined prevention therapy (vaginal estrogen, continuous prophylaxis, or postcoital prophylaxis), 7 (50%) had at least 1 UTI at mean 5.7 months (range, 2–7) after the initial visit. These patients were treated with self-start antibiotics, with 3 to 5 days of antibiotics based on prior culture susceptibility pattern, if available.

**Figure 3.**
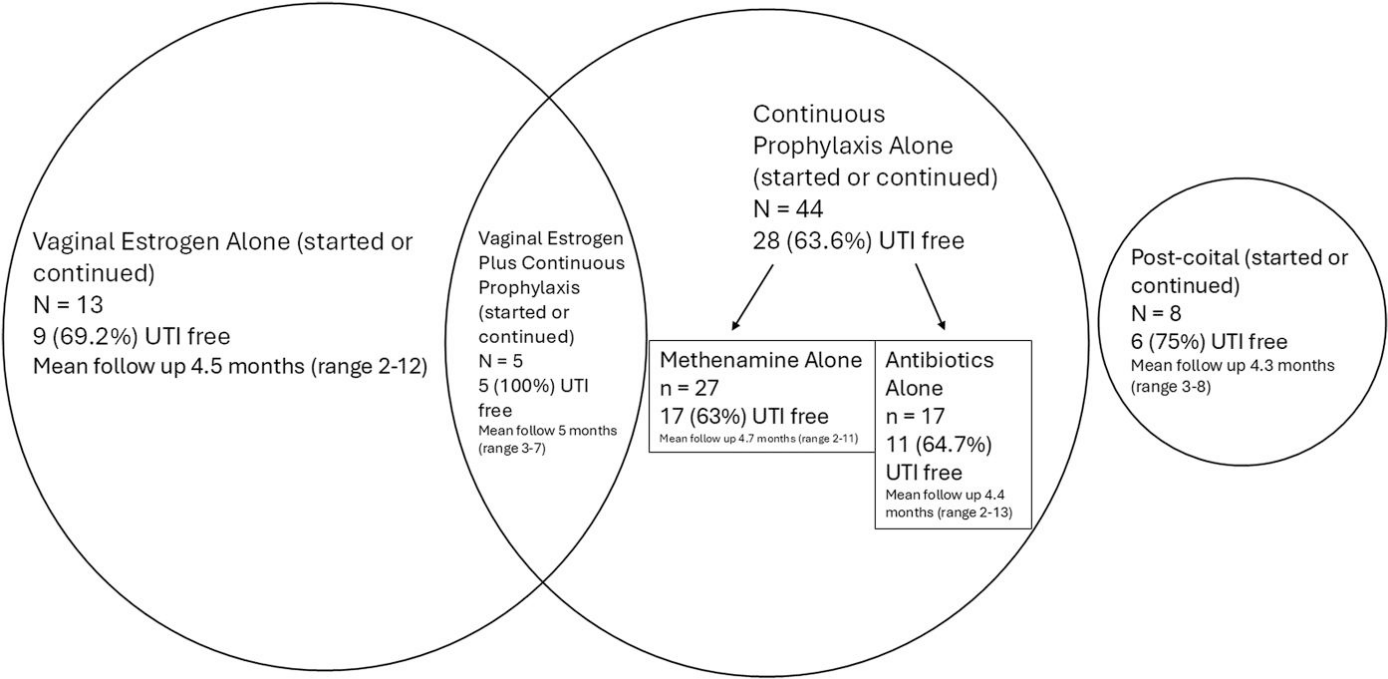
Prevention of UTIs for patients with recurrent/reinfections. UTI, urinary tract infection.

Of the 70 patients who accepted prevention therapy, 48 (69%) were UTI free at a mean follow-up of 4.6 months (range, 2–13; Figure 3). Individual efficacy was highest for vaginal estrogen plus continuous prophylaxis (100%), followed by postcoital prophylaxis (75%), vaginal estrogen alone (69%), antibiotics alone (65%), and methenamine hippurate with vitamin C alone (63%).

### Bacterial Persistence: Surgical and Medical Outcomes

We identified 20 (8%) patients with bacterial persistence, and a source was identified in 19 (95%), most commonly due to infected kidney or bladder stone (50%). The 1 patient with persistence without a known source was lost to follow-up. Surgical correction was performed in 8 (40%) patients leading to cure of their bacterial persistence, including stone removal (n = 6), removal of artificial urinary sphincter (n = 1), and excision of urethral sling sutures (n = 1). One patient with bacterial persistence expired due to cardiogenic shock. The remaining patients have been treated with long-term antibiotic suppression therapy.

### Role of Cystoscopy

Of note, of the 237 patients referred, most (n = 151, 64%) had a cystoscopy in their lifetime. Of those patients, 89 (59%) had a cystoscopy before the clinic visit, 19 (13%) after the visit, and 43 (28%) before and after the visit. Of the 151 patients, only 7 (5%) of cystoscopies found abnormalities that could contribute to UTIs: cystitis cystica (n = 2), bladder neck contracture (n = 2), urethral strictures (n = 2), and benign prostatic hyperplasia (n = 1).

### MDRO Data in Urine Cultures

A total of 60 patients (25%) had at least 1 MDRO, defined as a pathogen resistant to at least 1 antibiotic in ≥3 classes, as isolated in urine culture; of which, 77% were patients with complicated urologic tracts (Supplementary Table 3) [30]. Thirty percent of patients with at least 1 urine MDRO had a noninfectious syndrome after evaluation (ASB or LUTS, not UTI). Of the 60 patients with at least 1 MDRO, 31 (52%) had recurrent/reinfection. Of those 31 patients, 26 (84%) started the prevention regimen (vaginal estrogen and/or continuous prophylaxis). Of the 5 patients with MDROs who were prescribed vaginal estrogen, all were UTI free at a mean follow-up of 4.8 months (range, 3–8). Of the 10 patients with MDROs who were prescribed methenamine, 7 (70%) were UTI free at a mean follow-up of 4.2 months (range, 2–7).

### Impact of Clinic Management on Clinical Outcomes and Antimicrobial Utilization

Hospitalizations, emergency department visits, and urgent care visits were recorded for all 237 patients in this study. Admissions for planned surgical procedures and labor and delivery were excluded. We compared 1 year prior to the initial clinic visit vs 1 year after or, for bacterial persistence, 1 year after source control. Overall, the number of hospitalizations and emergency department/urgent care visits were significantly reduced (Table 2). Statistically significant reductions in hospitalizations were found in ASB, isolated UTIs, recurrent/reinfection, bacterial persistence with source control, and surgical prophylaxis. Of note, patients with bacterial persistence without source control did not show significant differences in hospitalizations postvisit. Statistically significant reductions in emergency department/urgent care visits were seen in all groups except isolated UTI and surgical prophylaxis. We also analyzed admissions and emergency department/urgent care visits that were not for UTIs in the same time frame and found no statistically significant difference, suggesting that our findings were due to the true impact of clinic management and not a result of the placebo effect. The average costs of a hospital admission and emergency department/urgent care visit for UTI at our institution are approximately $11 800 and $1600, respectively. Therefore, based on our data, we approximate that the clinic would effect $1.1 million in savings annually.

**Table 2.** Outcomes and Antibiotic Data.

	No. of Hospital Admissions for UTI			No. of Urgent Care/ED Visits for UTI		
Diagnostic Group	Previsit	Postvisit	*P* Value	Previsit	Postvisit	*P* Value
All patients (n = 237)	124	33	<.001	220	71	<.001
Asymptomatic bacteriuria (n = 41)	13	4	.029	24	6	.001
LUTS (n = 22)	2	2	>.99	17	7	.0412
UTI						
Isolated (n = 22)	10	2	.021	16	8	.102
Intermittent (n = 17)	1	2	.564	32	9	<.001
Recurrent/reinfection (n = 89)	52	13	<.001	109	35	<.001
Bacterial persistence						
With source control (n = 8)	16	2	<.001	2	0	.157
Without source control (n = 12)	7	6	.782	9	3	.833
Surgical prophylaxis (n = 15)	23	2	<.001	3	3	>.99
Other (n = 11)	0	0	…	8	0	.005

	Antibiotic Courses, Median (IQR)			Antibiotic Days of Therapy, Median (IQR)		
	Previsit	Postvisit	*P* Value	Previsit	Postvisit	*P* Value
All patients (n = 161)	4 (3–6)	2 (1–3)	<.001	30 (17–56)	14 (5–35)	<.001
Asymptomatic bacteriuria (n = 39)	4 (2–6)	1 (0–2)	<.001	28 (17–48)	5 (0–15.5)	<.001
LUTS (n = 15)	3 (2–4)	1 (0–1)	.001	17 (13–34)	5 (0–12.5)	.031
Recurrent/reinfection (n = 84)	4 (3–6)	2 (1–4)	<.001	34 (18–64.3)	17 (7.8–43)	.09
Bacterial persistence (n = 20)	4.5 (3–7.3)	2 (2–3.3)	.002	40 (21.8–68.5)	35 (15.8–77.8)	.98
Self-start (n = 14)^a^	3 (2–4)	1 (1–3)	.007	24.5 (15.8–33.8)	13 (7.8–35.3)	.217

Outcomes and antibiotic use were evaluated 1 year prior to and following the date of the initial clinic visit or source control, if applicable (eg, surgery, stone removal, urethral sling trim). Not all participants were included in the antibiotic analysis given the lack of antibiotic data available in the electronic medical record.

Abbreviations: ED, emergency department; LUTS, lower urinary tract symptoms; UTI, urinary tract infection.

^a^Self-start also represented in other groups.

Evaluation of antibiotic utilization was performed for the 161 patients with available data by stratification via diagnostic categories as outlined in Table 2. As compared with 1 year prior, 87% of patients received fewer antibiotic courses and 83% received fewer overall days of antibiotic therapy in the year following their initial clinic visit or, for those patients with bacterial persistence, from the date of source control. Median antibiotic exposure (ie, days of antibiotic therapy) was reduced from 30 to 14 days (*P* < .001). At the individual patient level, statistically significant reductions were observed in antibiotic courses and days of antibiotic therapy received. Across most diagnostic subgroups, courses of antibiotics received were significantly reduced following the initial specialist clinic visit. Statistically significant reductions in overall antibiotic days of therapy were observed for patients with ASB and LUTS. Of note, not all patients in each category were represented given the lack of electronic medical record data of antibiotic therapy, and antibiotics administered during certain admissions may not have been captured if patients were admitted to a hospital outside our electronic medical record.

Previsit antibiotic allergy or intolerance to antibiotics was present in 108 (46%), with multiple intolerances in 39 (16%). Of those with ASB and LUTS, previsit allergy or intolerance to antibiotics was observed in 18 (44%) and 11 (50%), respectively. However, antibiotic adverse effects were low in our cohort. No patients had intolerance or adverse effects due to vaginal estrogen. Of the 31 patients who started or continued methenamine, only 1 (3%) had to decrease the dose to daily administration due to gastrointestinal side effects. Of the 36 patients prescribed long-term antibiotic suppression or prophylaxis, only 1 (3%) had to stop due to side effects (fosfomycin gastrointestinal side effects). No patient had incident allergic reactions to prophylaxis or suppression. Of the 36 patients prescribed long-term suppressive antibiotics (cefpodoxime and cefadroxil), 2 (6%) developed toxin-positive symptomatic *Clostridioides difficile* infection, and both recovered with treatment.

Of all 237 patients seen, 228 (96%) survived to 1 year postvisit. Nine patients (4%) died, none of which had attributable mortality to UTI, and the causes of death were as follows: metastatic prostate cancer, metastatic urothelial cancer, central nervous system hemorrhage, metastatic bladder cancer, metastatic colon cancer, metastatic lung cancer, gastrointestinal bleeding, limb ischemia, and cardiogenic shock.

## DISCUSSION

UTIs affect approximately 50% to 60% of adult women and 20% of men in their lifetime, yet significant gaps in UTI management include a lack of standardized diagnostic definitions for categorizing complicated cases, uncertainty surrounding optimal antibiotic use (particularly in those with noninfectious syndromes), a lack of preventative measures in patients with recurrent infections, and failure to identify etiologies of bacterial persistence [1–15]. We developed a novel UDF to address these gaps in UTI management.

The definition of complicated UTI among guideline societies and regulatory bodies is variable with several definitions including host factors and clinical presentation [6, 8, 31–33]. In 2025, the IDSA issued a public comment revising the definition of complicated UTI to include any UTI outside the bladder [34]. Our approach categorized cases as complicated if urinary tract abnormality predisposed patients to infection, such as those with urinary retention or infected kidney stones. Our classification did not affect our duration of treatment but did affect our diagnostic strategy, particularly when evaluating urinary bacterial persistence. Our data demonstrated that complicated cases also had clinical differences, as such patients were more likely to present with pyelonephritis, develop recurrent/reinfection, have higher rates of MDROs, and respond less well to continuous prophylaxis.

ASB occurs in 1% to 6% of premenopausal women, up to 22% in postmenopausal women, and 10% of men aged >80 years [11, 12, 15, 35]. Evidence suggests that treatment of ASB leads to an increased risk of symptomatic UTI and up to 74% of patients with ASB receive inappropriate antibiotics, leading to longer hospital stays and higher rates of *C difficile* infection [14, 36]. Many patients are prescribed antibiotics for nonspecific complaints, such as fatigue, urine consistency and odor changes, confusion, incontinence, nausea, urinary retention, urinary catheter pain, and vaginal symptoms [15]. Our UDF adhered to a strict set of criteria to qualify for infections that did not include the aforementioned symptoms to qualify for a UTI diagnosis; as a result, 32% of patients referred with a diagnosis of UTI in actuality had noninfectious syndromes (usually ASB or LUTS). In addition, we relied on objective signs of infection (fever or unexplained sepsis) in patients who were nonverbal; undergoing intermittent catheterization; presenting with spinal cord injury; or harboring indwelling urinary catheters, ileal conduits, or neobladders, which allowed us to reduce the antibiotic burden in many of our patients with complex medical and surgical histories. This conflicts with the 2009 IDSA guidelines, which state that patients with catheters or spinal cord injuries with UTIs can present with malaise, altered mental status, lethargy, increased spasticity, autonomic dysreflexia, and sense of unease—none of which we included as symptoms of UTIs based on literature citing their poor specificity for UTIs [14, 17–19, 25,26, 28, 37].

We continued to follow these patients whom we categorized as having ASB or LUTS. According to our framework, patients with ASB showed significant reductions in hospitalizations, outpatient visits, and antibiotic utilization with no increased attributable mortality to UTIs—empowering clinicians and patients to refrain from antimicrobials when chief complaints consist solely of suprapubic pain, painless hematuria, altered mental status, malaise, lethargy, cloudy urine, foul-smelling urine, urinary retention, incontinence, dysreflexia, and spasms. Our intervention will help combat the growing threat of antibiotic resistance and *C difficile*. Indeed, our cohort of patients with noninfectious syndromes had an MDRO urine colonization rate of 28% and an antibiotic allergy and/or intolerance rate of 44% to 50%, further underscoring the urgent need to reduce antibiotic use in these patients.

Patients with recurrent/reinfections present a challenge to many health care providers. Our goal was to utilize an antibiotic-sparing regimen to prevent the development of resistance, spare the normal gastrointestinal flora, and minimize long-term adverse effects. For postmenopausal women with recurrent/reinfection, we found that the efficacy of vaginal estrogen was 69%, consistent with previous reviews [38]. The efficacy of methenamine hippurate as monotherapy for prophylaxis in our study was 63%, with 71% of failures in patients with complicated urologic tracts. Our findings are consistent with prior studies, although rigorous randomized controlled trials with strict definitions of UTIs are required [39]. Continuous antimicrobial prophylaxis was offered to patients who had breakthrough UTIs or for those who declined or could not tolerate vaginal estrogen and/or methenamine. Of the 17 patients prescribed antimicrobial prophylaxis as monotherapy, 65% were UTI free at follow-up. Data for efficacy of continuous antibiotic prophylaxis for UTIs are variable and range from 40% to 85%, and studies have shown up to 5% risk of development of resistance and infection with *C difficile* [40, 41]. Of note, the presence of an MDRO did not appear to affect the efficacy of prophylaxis in our cohort.

We recommend methenamine over antibiotic continuous prophylaxis given the comparable efficacy and more favorable adverse effect profile. This is supported by our data demonstrating a 6% rate of *C difficile* infection in those undergoing long-term antibiotics, as compared with none in the methenamine group. Of those with recurrent infection who declined prevention therapy, 50% had a UTI at follow-up and were encouraged to reconsider prevention therapy.

Twenty patients (8%) in our cohort were diagnosed with urinary bacterial persistence due to repeated symptomatic and microbial relapse with the same bacterial strain. These patients underwent urologic evaluation including imaging, cystoscopy, and bacterial localization studies. Sources of infection included infected stones, an infected artificial urinary sphincter, an infected bladder sling, and infected retained wires used in urologic surgery. Cystoscopy is often done for patients with recurrent UTIs, and although we found that only 5% of all cystoscopies provided information relevant to infections, we feel that it should be considered as these abnormalities can usually be detected only with cystoscopy and can be frequently corrected. Our data showed a significant reduction in hospitalizations for patients with bacterial persistence who had source control—but not in those without source control, providing further evidence to remove infected foci of infection when found.

The impact of the UDF resulted in a statistically significant reduction of hospitalizations, emergency department/urgent care visits, and antibiotic use for UTIs in our patients. These data have several important implications. For patients with true infections (recurrence and persistence), our intervention of prophylaxis or surgical management reduced their need for admission, emergency department/urgent care visit, and antibiotics for UTIs and, by extension, their burden of infection. Second, we reduced antibiotic exposure in patients with noninfectious syndromes (ASB and LUTS), reducing their risk of development of resistance and other adverse effects from antimicrobials. Third, by reducing admissions, outpatient visits, and antibiotic use, we are employing cost-saving benefits for the patients and health care system at large, as estimated annual costs of prescriptions alone for UTIs reach up to US $1.6 billion [42]. Based on our 1-year data, we could save $1.1 million in health care annually. This approach can be implemented in many practice settings and institutions spanning academic centers and community hospitals, potentially leading to major effects in health care utilization.

Our study has limitations. Most of our patients were White females. Our data on prophylactic efficacy are limited as this was not a randomized controlled trial, and we had relatively low numbers for each group, limiting the power of the data. We also established efficacy of prophylaxis at different time points depending on the timing of clinic follow-up. For those who did not follow up, efficacy was based on chart review of positive cultures in our system. In addition, hospitalization and urgent care visits were limited to available records in our electronic medical record—it is possible that a patient sought health care elsewhere and this would not have been recorded, leading to possible false elevation in prophylaxis efficacy or reduction in health care visits. In addition, although we did show a reduction in hospitalizations and emergency department/urgent care visits, we did not have a control arm to compare.

In summary, utilization of a standardized interdisciplinary UDF can support clinicians in clarifying urinary concerns, eliminate unnecessary antibiotic treatment for patients without infections, reduce the infection rate in patients with recurrent infections, and identify and remove urologic foci that lead to bacterial persistence. The reduction of health care utilization for UTIs for all types of patients will ultimately reduce health care costs, improve antibiotic stewardship, and combat the growing threat of multidrug resistance.

## Supplementary Material

ofaf293_Supplementary_Data

## Supplementary Data

Supplementary materials are available at *Open Forum Infectious Diseases* online. Consisting of data provided by the authors to benefit the reader, the posted materials are not copyedited and are the sole responsibility of the authors, so questions or comments should be addressed to the corresponding author.
