# Supplementary material for: A Novel and Reproducible Urinary Diagnostic Framework Reduces Health Care and Antibiotic Utilization for Urinary Tract Infections
Source: Open Forum Infect Dis. 2025 May 15;12(6):ofaf293. doi: 10.1093/ofid/ofaf293 (PMC12125672; doi:10.1093/ofid/ofaf293)
Supplement: ofaf293_Supplementary_Data [file ofaf293_supplementary_data.docx]

| **Supplemental Table 1. Participant Characteristics.** | |  |  |  |
| --- | --- | --- | --- | --- |
| **Demographics** | **All Patients n = 237** | **Complicated n = 137** | **Uncomplicated n = 100** | ***p*-value** |
| Female sex | 164 (69.2%) | 80 (58.4%) | 84 (84%) | 0.03 |
| Male sex | 73 (30.8%) | 57 (41.6%) | 16 (16%) | 0.0007 |
| Age in years (mean (range) | 61.2 (19-94) | 62 (19-94) | 58.7 (21-92) | 0.17 |
| Racial and Ethnic Category |  |  |  |  |
| Arabic | 3 (1.3%) | 2 (1.5%) | 1 (1%) | 0.75 |
| Asian | 6 (2.5%) | 3 (2.2%) | 3 (3%) | 0.73 |
| Black | 30 (12.7%) | 16 (11.7%) | 14 (14%) | 0.65 |
| Hispanic | 40 (16.9%) | 26 (19%) | 14 (14%) | 0.38 |
| White | 158 (66.7%) | 90 (65.7%) | 68 (68%) | 0.84 |
| **Clinical Characteristics** |  |  |  |  |
| Charlson Score (Mean (Range)) | 3.3 (0-10) | 3.9 (0-10) | 2.6 (0-9) | 0.0001 |
| Co-morbidities |  |  |  |  |
| Cancer | 68 (28.7%) | 54 (39.4%) | 14 (14%) | 0.0005 |
| Prostate | 18 | 18 | 0 | 0.0003 |
| Bladder | 17 | 17 | 0 | 0.0004 |
| Renal | 4 | 3 | 1 | 0.5 |
| Other | 29 | 16 | 13 | 0.79 |
| Chronic kidney disease | 22 (9.3%) | 16 (11.7%) | 6 (6%) | 0.18 |
| Connective tissue disease | 13 (5.5%) | 8 (5.8%) | 5 (5%) | 0.81 |
| COPD | 6 (2.5%) | 3 (2.2%) | 3 (3%) | 0.73 |
| Coronary artery disease | 16 (6.8%) | 9 (6.6%) | 7 (7%) | 0.91 |
| CVA/TIA | 14 (5.9%) | 8 (5.8%) | 6 (6%) | 0.95 |
| Diabetes mellitus | 45 (19%) | 30 (21.9%) | 15 (15%) | 0.26 |
| Heart failure | 8 (3.4%) | 4 (2.9%) | 4 (4%) | 0.68 |
| Hemiplegia | 6 (2.5%) | 6 (4.4%) | 0 (0%) | 0.036 |
| Liver disease | 6 (2.5%) | 5 (3.6%) | 1 (1%) | 0.23 |

| **Supplemental Table 2. Anatomic Characterization of the Participant’s Urinary Tract.** |  |
| --- | --- |
|  |  |
| **Uncomplicated** | 100 (42.2%) |
|  |  |
| **Complicated** | 137 (57.8%) |
| **Urethra** |  |
| Stricture | 6 |
| Flayed | 1 |
| Congenital duplication | 1 |
| Urethrovaginal fistula | 1 |
| Urethro-cutaneous fistula | 1 |
| Mid-urethral sling | 1 |
| Short urethra | 1 |
| Urethral adenocarcinoma | 1 |
|  |  |
| **Bladder** |  |
| Bladder cancer | 17 |
| Cystectomy | 14 |
| Neurogenic bladder | 12 |
| Vesicoureteral reflux | 4 |
| Cystitis cystica | 3 |
| Colo-vesicular fistula | 2 |
| Bladder augmentation | 2 |
| Bladder sling | 2 |
| Bladder suspension | 1 |
| Bladder exstrophy | 1 |
| Bladder calculi | 1 |
|  |  |
| **Prostate** |  |
| Prostate cancer | 18 |
| Prostatectomy | 8 |
| Radiation therapy | 13 |
| ADT | 3 |
| Chemotherapy | 4 |
| TURP | 2 |
| Urinary retention | 3 |
| Obstructive BPH | 7 |
| Prostatectomy | 9 |
| Simple | 1 |
| Radical | 8 |
|  |  |
| **Kidney/Ureter** |  |
| Nephrolithiasis | 23 |
| Renal transplant | 15 |
| Nephrostomy tube | 8 |
| Hydronephrosis | 7 |
| Ureteral stent | 3 |
| UPJ Obstruction | 3 |
| Nephrectomy | 2 |
| Ureteral stricture | 1 |
| Tortuous ureter | 1 |
| Retained ureter wire | 1 |
|  |  |
| **Penile** |  |
| Hypospadias | 4 |
|  |  |
| **Pregnancy** | 4 |
|  |  |
| **Other**^a^ | 13 |

^a^Others include urinary retention of other cause (11), gender reaffirming surgery (orchiectomy/vaginoplasty, 1), urachal cyst (1).

| **Supplemental Table 3. Urinary multidrug-resistant organisms in our cohort.** | |
| --- | --- |
| n = 60 (25.3%) |  |
| Anatomic Categorization |  |
| Complicated | 46 (76.7%) |
| Uncomplicated | 14 (23.3%) |
| Final Diagnosis |  |
| Urinary tract infection | 36 (60%) |
| Non-infectious syndrome | 18 (30%) |
| Surgical prophylaxis | 6 (10%) |
| Type of MDRO^a^ |  |
| ESBL | 54 |
| *Escherichia coli* | 40 |
| *Klebsiella pneumoniae* | 11 |
| *Enterobacter cloacae* | 1 |
| *Citrobacter freundii* | 1 |
| *Providencia stuartii* | 1 |
| CRE | 1 |
| *K. pneumoniae* (KPC) | 1 |
| VRE | 8 |
| MRSA | 3 |
| Carbapenem-resistant *Pseudomonas aeruginosa* | 2 |

^a^Extended spectrum beta-lactamase (ESBL). Carbapenem resistant Enterobacterales (CRE). Vancomycin-resistant Enterococci (VRE). Methicillin-resistant *Staphylococcus aureus* (MRSA).
